# Salicylic Acid as a Prophylactic against Cholera

**Published:** 1885-01

**Authors:** 


### Salicylic Acid as a Prophylactic Against Cholera.

In a letter published in the *Bulletin gen. de Therap.*, Dr. Beaudon says that his experience with salicylic acid as a disinfectant has been so satisfactory as to lead him to suggest that the drug be generally used as a prophylactic in case a country is threatened with cholera. He proposes the following rules for its use: 1. Make a solution of ten grammes of the acid in two hundred grammes of alcohol; add a teasponful of this solution to a glass of water. 2. Bathe, wash the hands, and rinse out the mouth several times a day with this mixture. Let all flannel and woolen clothing be impregnated with it. These precautions apply particularly to physicians, who are exposed to the contagion. 3. Take a teaspoonful of this same solution with each meal. The objection may perhaps be raised, he says, that the microbe (if there is a microbe) will be taken in by the respiratory passages. He does not discuss this idea, but limits himself to the statement that whatever may be the channel of introduction of the microbe, it will not find a very fertile field for development if the individual has used and still uses a substance which opposes the entrance and propagation of the poison.—*N. Y. Med. Journal.*

